# The Arsenal of Bioactive Molecules in the Skin Secretion of Urodele Amphibians

**DOI:** 10.3389/fphar.2021.810821

**Published:** 2022-01-14

**Authors:** Ana L. A. N. Barros, Abdelaaty Hamed, Mariela Marani, Daniel C. Moreira, Peter Eaton, Alexandra Plácido, Massuo J. Kato, José Roberto S. A. Leite

**Affiliations:** ^1^ Núcleo de Pesquisa em Morfologia e Imunologia Aplicada, NuPMIA, Faculdade de Medicina, Universidade de Brasília, Brasília, Brazil; ^2^ Programa de Pós-graduação em Medicina Tropical, PPGMT, Núcleo de Medicina Tropical, NMT, Faculdade de Medicina, UnB, Brasília, Brazil; ^3^ Instituto de Química, IQ, Universidade de São Paulo, São Paulo, Brazil; ^4^ Chemistry Department, Faculty of Science, Al-Azhar University, Nasr City-Cairo, Egypt; ^5^ IPEEC-CONICET, Consejo Nacional de Investigaciones Científicas y Técnicas, Puerto Madryn, Argentina; ^6^ LAQV/REQUIMTE, Departamento de Química e Bioquímica, Faculdade de Ciências da Universidade do Porto, Porto, Portugal; ^7^ Joseph Banks Laboratories, The Bridge, School of Chemistry, University of Lincoln, Lincoln, United Kingdom; ^8^ Bioprospectum, Lda, UPTEC, Porto, Portugal

**Keywords:** amphibians, urodela, bioactive molecules, peptides, alkaloids

## Abstract

Urodele amphibians (∼768 spp.), salamanders and newts, are a rich source of molecules with bioactive properties, especially those isolated from their skin secretions. These include pharmacological attributes, such as antimicrobial, antioxidant, vasoactive, immune system modulation, and dermal wound healing activities. Considering the high demand for new compounds to guide the discovery of new drugs to treat conventional and novel diseases, this review summarizes the characteristics of molecules identified in the skin of urodele amphibians. We describe urodele-derived peptides and alkaloids, with emphasis on their biological activities, which can be considered new scaffolds for the pharmaceutical industry. Although much more attention has been given to anurans, bioactive molecules produced by urodeles have the potential to be used for biotechnological purposes and stand as viable alternatives for the development of therapeutic agents.

## Introduction

The skin of amphibians exerts a broad spectrum of functions, which are fundamental for their homeostasis and interaction with the environment. Different multicellular skin glands produce compounds involved in both vital processes and defense strategies (Clarke, 1997; Heiss et al., 2009). Mucous glands produce a slippery mucus film, made basically of mucopolysaccharides and mucoproteoglycans, which minimize underwater friction and also make the animal slippery to predators. Other functions of the mucus include the prevention of water loss and the maintenance of a moist surface for skin gas exchange when the animal leaves the water. Granular glands are classified into different types depending on their histological characteristics and, on the other hand, synthesize and release compounds (e.g., amines, peptides and alkaloids) that participate in several defense mechanisms against potential aggressors, which can be either large predators or pathogenic microorganisms (Bucciarelli et al., 2016; Demori et al., 2019). In the first case, the compounds might irritate mucous membranes, cause an unpleasant sensation (e.g., pain or distastefulness), or even be highly toxic and lethal for their aggressors. In the second case, antimicrobial substances, in association with the commensal microbiota, prevent the colonization or infection by fungi, bacteria, and viruses (Brunetti et al., 2021).

The skin secretions of amphibians have been historically used as ethno-pharmaceutical drugs in a variety of cultures. Extensive research on this topic has accumulated over the years and many of the molecules secreted by amphibians have been isolated and characterized to date. These were found to influence different processes in living systems, acting, for example, as myotropic, immunomodulatory, antibiotic, anti-inflammatory, and antioxidant compounds. For this reason, the skin of amphibians has been recognized as a storehouse of bioactive molecules with pharmacological potential. In this context, “bioactivity” refers to the ability of a molecule to exert a given effect in an organism that can be explored for biotechnological applications (e.g., food or pharmaceutical industries), regardless of its original (evolutionary) function in its source. Indeed, amphibian-derived bioactive molecules currently stand as possible substitutes for conventional drugs or as drug leads for the development of therapeutic agents. Among amphibian orders, much more attention has been given to anurans than to that given to caecilians and urodeles in the research of bioactive molecules.

The Urodela order comprises salamanders and newts currently distributed in North and Central America, Europe, Asia, and North Africa, with some species occurring in South America (Baitchman and Herman, 2015). The order is divided into Salamandroidea and Cryptobranchoidea suborders (Jia and Gao, 2016). Salamandroidea differs from its sister clade Cryptobranchoidea regarding anatomic characteristics as bones jaw and ribs, and internal fertilization, and includes the genus *Amphiuma*, *Ambystoma*, *Plethodon*, *Salamandra*, *Tylotriton*, and *Taricha* (Anderson, 2012; Jia and Gao, 2016). Like anurans, bioactive molecules produced by urodeles have the potential to be used for biotechnological purposes, which make this group of amphibians a promising alternative to develop products with application in human health (Afolabi et al., 2018). In this mini review, we provide a brief appraisal of molecules isolated from the skin of urodele amphibians with an overview of their biological activities, with emphasis on peptides and alkaloids.

## Alkaloids

Alkaloids have been isolated from phylogenetically diverse organisms, including microorganisms (Zhang et al., 2012; Casciaro et al., 2019), plants (Lelario et al., 2019; Petruczynik et al., 2019), and animals (Dumbacher et al., 2000; Jones et al., 2012; Santos et al., 2012; Saporito et al., 2012; Cushnie et al., 2014; Jeckel et al., 2015; Ligabue-Braun and Carlini, 2015; Knepper et al., 2019). Alkaloids have been shown to possess important biological activities as a defense mechanism against microorganisms and predators (De Luca and Laflamme, 2001; Zhang et al., 2021). They are generally derived from dietary sources of these animals (Macfoy et al., 2005), but certain alkaloids, such as samandarins of salamanders, are synthesized *de novo* from cholesterol (Habermehl and Haaf, 1968; Daly et al., 2005).

**FIGURE 1:**
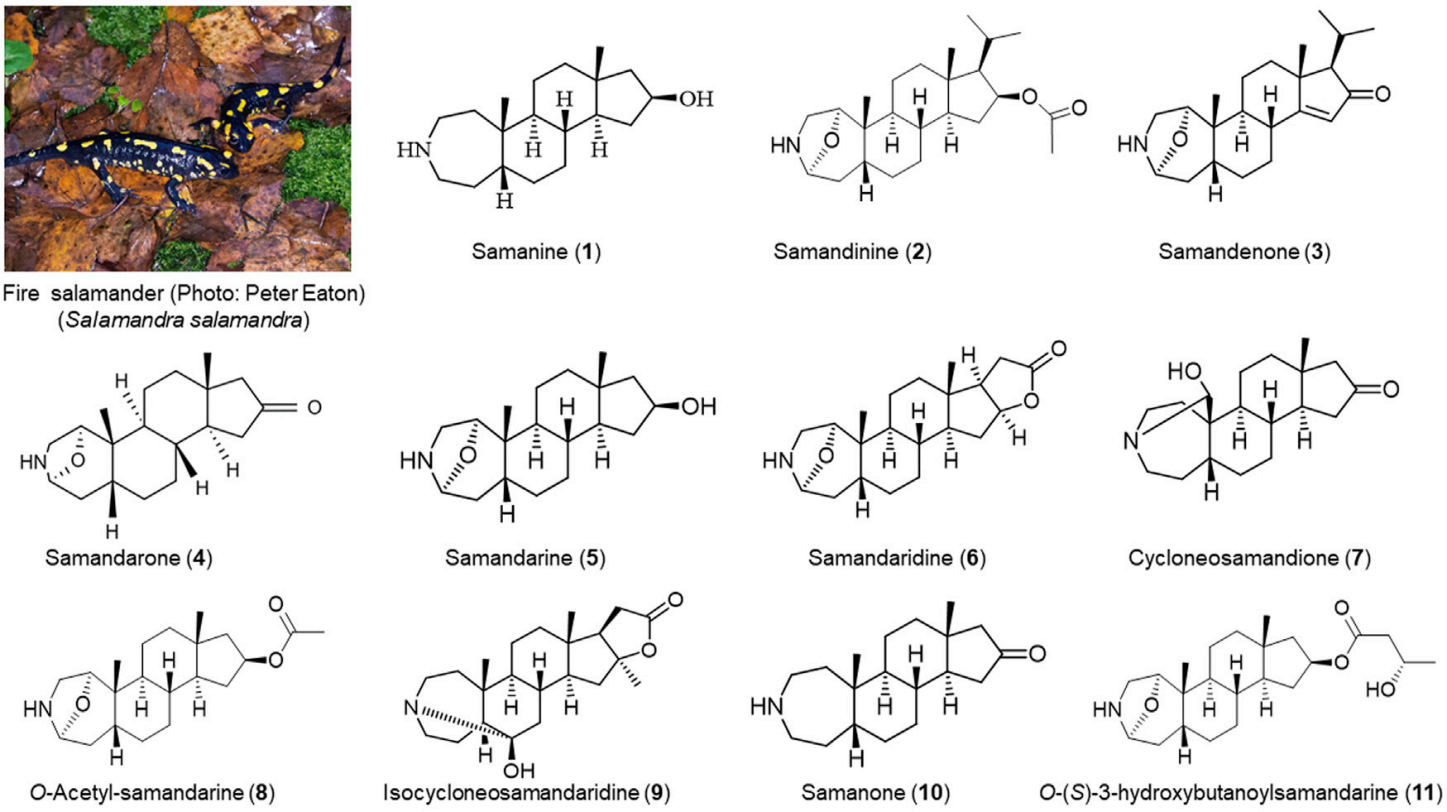
Alkaloids identified in the skin secretion of fire salamander (*Salamandra salamandra*). **(1)**: (Habermehl, 1967; Habermehl and Haaf, 1968; Habermehl and Preusser, 1969; Habermehl, 1971); **(2)** (Habermehl and Vogel, 1969); **(3**,**4**,**5**,**6**, and **7)**: (Habermehl, 1964, Habermehl, 1967; Habermehl and Haaf, 1968; Habermehl and Preusser, 1969; Habermehl, 1971); **(8)** (Habermehl, 1964); **(9)** (Daly et al., 2005); **(10**,**11)** (Knepper et al., 2019).

The search for alkaloids in amphibians of the Urodela order was pioneered by Zalesky in 1866, who isolated an alkaloid named samandarine from fire salamander (*Salamandra salamandra*) secretion. Then, Schöpf and Habermehl described a series of steroidal alkaloids and elucidated their absolute configurations (Weitz and Wolfel, 1962; Habermehl, 1967; Knepper et al., 2019). To date, the alkaloids characterized from the skin secretion of fire salamanders include samanine (**1**), samandinine (**2**), samandenone (**3**), samandarone (**4**), samandarine (**5**), samandaridine (**6**), cycloneosamandione (**7**), *O*-Acetyl-samandarine (**8**) and isocycloneosamandaridine (**9**) (Habermehl, 1964; Habermehl, 1967; Habermehl and Vogel, 1969; Habermehl, 1971; Daly et al., 2005), samanone (**10**) and *O*-(*S*)-3-hydroxybutanoylsamandarine (**11**) (Knepper et al., 2019) (Figure 1). A study of toxins using gas chromatography/mass spectrometry confirmed the presence of samandarine and/or samandarone steroidal alkaloids in all species of *Salamandra* as well as in representatives of *Lyciasalamandra* group. Other salamandrids, such as *Calotriton, Euproctus, Lissotriton*, and *Triturus*, also present low concentrations of samandarone (Vences et al., 2014), evidencing the widespread of alkaloids in the skin secretion of this group of animals.

Antibiotic resistance has become one of the major concerns for global health systems and the worldwide emergence of microbial resistance to available antibiotic drugs has led to an exhaustive search for new molecules with antimicrobial properties, and those from urodeles are valid alternatives. The alkaloids from the secretion of *S. salamandra* were able to inhibit the growth of fungi (*Geotrichum candidum*) and bacteria (*Bacillus subtilis*) (Habermehl and Preusser, 1969). Samandarone completely inhibited the growth of fungi (*Phycomyces blakesleeanus*) at a minimum effective concentration of 3 × 10^−6^ M. Samandarone and salamandarine from *S. maculosa* inhibit the growth of *Escherichia coli, Proteus mirabilis, Bacillus subtilis, Phycomyces blakesleeanus, Saccharomyces cerevisiae, Geotrichum candidum,* and *Trichoderma viride* in agar-diffusion tests. Samandarone presented the widest zones of inhibition for all the strains and also inhibited the growth of *Penicillium expansum*. Samandarone, samandarine, and samandaridine completely inhibited the growth of *Saccharomyces cerevisiae* at a minimum effective concentration of 1.5 × 10^−6^ M (Preusser et al., 1975). Although the performance of alkaloids as antibiotics against a variety of microorganisms stand out, their toxicity in major groups of vertebrates have also been documented discouraging its pharmacological use (von Byern et al., 2017). Indeed, alkaloids from urodele amphibians are known to be neurotoxins towards vertebrates (Phisalix, 1900; Mebs and Pogoda, 2005; Erjavec et al., 2017; Preißler et al., 2019).

## Peptides

Peptides form another group of molecules with antibiotic activity produced by the skin of salamanders and newts, but with presumed (and in some cases, proven) low toxicity (Peters et al., 2010). Among these, antimicrobial peptides (AMPs) are found in several species (Jenssen et al., 2006). In general, antimicrobial peptides have between 12 and 100 amino acid residues, are cationic, amphipathic and have a range of typical secondary structures, as α-helices, β-sheets, and extended and flexible loops (Nguyen et al., 2011; Gao et al., 2017). The development of resistance by microorganisms to AMPs is considered relatively unlikely, mainly because they target the cell membrane directly. It has also been claimed that they have a poorly mutable structure, and present a wide range of epitopes, which makes their recognition difficult (Yeaman and Yount, 2003; Shen et al., 2018). Therefore, AMPs have been considered as alternative candidates for the development of new antimicrobial agents either alone (Nicola et al., 2019) or in combination with conventional antibiotics (Choi and Lee, 2012; Nuding et al., 2014).

In amphibians of the Urodela order, peptides with potential biological activities were isolated particularly from the skin secretion, although also from different parts of the animals (Table 1 **)** (Pei et al., 2018). The peptide andricin 01 was isolated from the skin secretion of giant Chinese salamander *Andrias davidianus*. Andricin 01 showed antimicrobial activity against Gram-negative and Gram-positive bacteria however, it did not show cytotoxic activity to human hepatocytes or renal cells, and no hemolytic activity was observed (Pei and Jiang, 2017).

**TABLE 1:** Primary structure of peptides isolated from urodele amphibians.

Source	Name	Structure	Molecular Mass	Biological activity	Reference
*Andrias davidianus*	Andricin 01	AIGHCLGATL	955.1 Da	Antibacterial	Pei and Jiang, (2017)
*Andrias davidianus*	Andricin B	GLTRLPSVIK	-	Antibacterial Antifungal	Pei et al. (2018)
*Plethodon cinereus*	F15	-	13.75 kDa	Antibacterial	Fredericks and Dankert, (2000)
*Cynops fudingens*	CFBD-1	FAVWGCADYRGYCRAACFAFEYSLGPKGCTEGYVCCVPNTF	4,251.37 Da	Antibacterial	Meng et al. (2013)
*Salamandra salamandra*	Salamandrin-I	FAVWGCADYRGY-NH2	1,406.6 Da	Antioxidant	Plácido et al. (2020)
*Cynops pyrrhogaster*	NVRP-1	HSDAVFTDNYSRLLGKTALKNYLDGALKKE	3,000 Da	Muscle relaxant	Teranishi et al. (2004)
	NVRP-2	HSDAVFTDNYSRLLAKTALKNYLDGALKKE			
	NVRP-3	HSDAVFTDNYSRLLGKIALKNYLDEALKKE			
	NVRP-4	HSDAVFTDNYSRLLGKTALKNYLDSALKKE			
*Tylototriton verrucosus*	Tylotoin	KCVRQNNKRVCK	1,473.80 Da	Wound healing	Mu et al. (2014)

The 13.75 kDa peptide F15 was isolated from the skin of the red-backed salamander *Plethodon cinereus*. In a colony counting assay, F15 reduced *S. aureus* by 90% in 2 h, thus showing strong antibacterial activity against Gram-positive bacteria (Fredericks and Dankert, 2000). Another work identified CFBD-1 in the skin of the newt *Cynops fudingens*, a peptide of the β-defensin class, consisting of 41 amino acids, with a molecular mass of 4251.37 Da. This defensin was shown to be active mainly against the Gram-positive bacteria *S. aureus*, similarly to the previously described F15 peptide, with a minimum inhibitory concentration (MIC) of 65 μg/ml (Meng et al., 2013).

As a first approximation, numerous colleagues determined the antimicrobial activity of the total crude extract or the protein fraction of the skin secretion of several urodels. A study performed with the analysis of the total number of peptides isolated from the skin of salamander larvae and adults of the *Ambystoma tigrinum* species revealed antibacterial activity against *Bacillus dendrobatidis*, *Staphylococcus aureus,* and *Klebsiella* sp. (Sheafor et al., 2008). Studies with the crude extract of the cutaneous secretions of the species *Lissotriton vulgaris* and *Triturus ivanbureschi* showed that these secretions present a total of 18 and 20 protein fractions, respectively. These extracts showed antimicrobial activity for several bacteria, *Escherichia coli* and *Enterococcus faecalis*, and fungi, *Candida albicans*, strains, and hemolytic activity for human and rabbit red blood cells (Mert et al., 2018). The antimicrobial activity may be is a result of the action of active peptides (Pukala et al., 2006). In addition, they also showed high cytotoxic effects for cancer cell lines, with the highest anti-cancer activity of both secretion samples (*L.vulgaris* and *T. ivanbureschi*) being for the MDA-MB-231 breast cancer cell line (Mert et al., 2018). These preliminary findings highlight the importance of further research on the skin secretions of these animals, to characterize the molecules responsible for the antibiotic activities.

In addition to AMPs, other peptides isolated from urodeles can also have diverse pharmacological properties, such as antioxidant, and immunomodulatory activities (Coorens et al., 2017; Holthausen et al., 2017; Lee et al., 2019; Woodhams et al., 2020). Some examples of the last one is the neutralization of endotoxins, chemotaxis, and wound healing activities (Mangoni and Casciaro, 2020).

Reactive oxygen species (ROS) are necessary for the normal biochemical processes of cells (Litescu et al., 2011). However, in excessive amounts, when oxidative stress occurs, they can damage some structures, like DNA, proteins, and lipids (Duarte and Lunec, 2005). This process is related to cardiovascular disorders, diabetes, neurodegenerative diseases, cancer, chronic inflammatory diseases (Llesuy et al., 2001).

Synthetic chemical antioxidants often have low stability, cytotoxic, and carcinogenic effects, which led to search for natural antioxidants with low cytotoxicity (Gürlek et al., 2020). Salamandrin-I is a recently described molecule that showed relevant antioxidant activity. This molecule was the first peptide identified in the skin of the European fire salamander (*S. salamandra*). The peptide neutralizes the free radicals DPPH and ABTS at nontoxic concentrations for microglial cells and human red blood cells (Plácido et al., 2020). The discovery of this peptide paves the way for further investigation of other antioxidant peptides in the skin secretion of urodeles.

The medication currently available for wound healing is costly, has low activity, and produces hyperplastic scars necessitating the search for new drugs (Hardwicke et al., 2008). The amphibian peptides have shown potential as alternative treatment (Cao et al., 2018). The peptide tylotoin identified from the salamander *Tylototriton verrucosus* skin has shown to be effective in assay with dermal wounds in mice. It showed a similar wound healing capacity as the epidermal growth factor. The peptide has been shown to promote increased motility and proliferation of keratinocytes, vascular endothelial cells, and fibroblasts, resulting in accelerated re-epithelialization and formation of granulation tissue at the wound site. Tylotoin promotes the release of transforming growth factor β1 (TGF-β1) and Interleukin 6 (IL-6), which are essential in the wound healing response (Mu et al., 2014).

In general, due to its distinct biochemical and therapeutic properties, peptides are under development to disrupt protein-protein interactions and target or inhibit intracellular molecules such as proteinaceous receptors (Lee et al., 2019). Moreover, numerous peptide molecules were described to interact with microbial membranes through different mechanisms, destabilizing them, making peptides a unique class of pharmaceutical compounds (Vineeth Kumar and Sanil, 2017). The field of peptide drug discovery has evolved from both computational-aided rational design and combinatorial chemistry to the discovery of new molecules as stepping stones to new drug design, involving academic groups and private companies (de la Torre and Albericio, 2020; Al Shaer et al., 2020; Sampaio de Oliveira et al., 2020). With a high level of dynamism several peptide drugs are approved for clinical use in the United States, Europe, and Japan, and near 400 peptides are already in clinical development (Lee et al., 2019). The urodel peptides deserve more attention to clarify their structural features and mechanism of action.

## Other Molecules

In addition to the alkaloids and peptides, other compounds have been described from salamanders and newts. CCK-TV is a molecule of the cholecystokinin class, a gastrointestinal hormone, identified from salamander skin *Tylototriton verrucosus*. CCK-TV showed potential for inducing muscle contraction isolated smooth striatum from the porcine gallbladder in concentrations range of 5.0 × 10^−11^ to 2.0 × 10^−6^ M (Jiang et al., 2015).

Dermal secretion analysis of salamander *Plethodon cinereus*, shows the presence of three fatty acids with antibacterial activity. The inhibition test showed that myristoleic acid, linolenic acid, and palmitoleic acid, inhibited the growth of *Bacillus cereus* in 24 h, at concentrations of 27, 7, and 6.9 µg, respectively, with halos of inhibition around 8.0–6.5 mm in diameter (Rickrode et al., 1986).

Another class of bioactive molecules found in amphibian skin are biogenic amines. Amines, such as putrescine, histamine, tryptamine, and phenylethylamine, are low molecular weight bases found in living organisms or as breakdown products of the fermentation process (Erspamer, 1971; Jovaisiene et al., 2017). Studies reported the presence of high concentrations of tryptamine and serotonin in the skin of salamander species (Erspamer, 1971; Roseghini et al., 1976; Luddecke et al., 2018).

## Conclusion

Understanding the Earth’s biodiversity is important for describing genetic diversity and prospecting for bioactive molecules from diverse organisms. Skin secretion of amphibians of order Urodela are a unique source of alkaloids, peptides, biogenic amines and other compounds with diverse structures and functions to be considered as bioactive compounds. The studies compile in this review shows that steroidal alkaloids and peptides from Urodela skin secretions are considered the first line of defense against pathogens, however diverse limitations of the studies performed as difficulties to obtain toxins or to synthetize it by artificial methods made that the full capacity of these unique molecules as antimicrobials has not been properly studied and most of the studies described are still preliminary. In the case of peptides, the availability of synthetic methodology has provided access to explore the structures of analogues while in case of complex molecules, the partial or total synthesis can be more challenging specially when only putative compounds are inferred from the mass spectrometric data and the number of chiral centers and the number of possible diastereoisomers makes the task even more complex.

Despite considerable knowledge on alkaloids from urodeles, studies of secretions in terms as-of-yet unidentified antimicrobial components are needed to improve the current understanding of the complex toxin system of their skin (Luddecke et al., 2018). Urodele-derived molecules are underexplored therapeutic alternatives to conventional antibiotics used to treat fungal and bacterial infections.
